# Longitudinal associations between group-sport-related physical activity and psychological distress in adolescents: the parallel indirect roles of self-esteem and perceived social support

**DOI:** 10.3389/fpsyg.2026.1880098

**Published:** 2026-09-03

**Authors:** Liyan Wu, Jie Pan, Jun Chen

**Affiliations:** 1 Faculty of Sports and Health, Wenzhou University, Wenzhou, China; 2 Wenzhou Foreign Language School, Wenzhou, China

**Keywords:** adolescents, group-sport-related physical activity, longitudinal study, perceived social support, psychological distress, self-esteem

## Abstract

**Background:**

This study examined prospective longitudinal associations among group-sport-related physical activity, self-esteem, perceived social support, and psychological distress among adolescents.

**Methods:**

A three-wave longitudinal study was conducted among 545 Chinese adolescents. Participants had a mean age of 14.9 years (SD = 1.6; range = 12–18 years). Group-sport-related physical activity dose, operationalized with the Physical Activity Rating Scale-3 (PARS-3), was assessed at T1; self-esteem and perceived social support were assessed at T1 and T2 (perceived social support was also assessed at T3); and psychological distress was assessed at T1 and T3. A temporally ordered parallel mediation model with baseline controls was estimated. Because not all constructs were measured at every wave, the design was not a complete panel and did not permit a random-intercept cross-lagged panel model (RI-CLPM).

**Results:**

Higher T1 PARS-3 scores were prospectively associated with lower T3 psychological distress. Self-esteem and perceived social support at T2 showed significant indirect associations, with the self-esteem pathway being comparatively stronger. Exploratory lagged analyses indicated that T1 self-esteem was associated with T2 perceived social support after accounting for prior support; however, these conventional lagged paths cannot be interpreted as within-person effects.

**Conclusions:**

Group-sport-related physical activity may be prospectively associated with lower adolescent psychological distress through psychological and social resources. Given the convenience sample, asymmetric measurement schedule, possible residual confounding, and absence of an RI-CLPM, the findings should be interpreted as prospective associations rather than definitive causal mechanisms.

## 1 Introduction

Adolescent mental health has become a major public health concern worldwide. According to the World Health Organization, approximately 14% of adolescents experience mental disorders, with depression and anxiety representing the most prevalent forms of psychological difficulties (Silva et al., 2020). In China, the mental health status of adolescents has attracted increasing attention in recent years (Wang et al., 2020). Epidemiological investigations have indicated that depressive symptoms are highly prevalent among Chinese middle and high school students (Zhou et al., 2024), while psychological distress among adolescents has been closely associated with academic pressure, interpersonal difficulties, and elevated parental expectations (Chyu and Chen, 2022). Given that mental health problems emerging during adolescence frequently persist into adulthood and are associated with substantial social and developmental consequences, identifying modifiable protective factors has become an important research priority.

Among the various factors associated with adolescent psychological adjustment, physical activity has consistently been identified as an important protective resource (Chen et al., 2024). Previous studies have demonstrated that adolescents who engage in regular physical activity report lower levels of depression and anxiety, higher levels of subjective well-being, and more positive self-evaluations (McMahon et al., 2017). Nevertheless, the psychological mechanisms through which physical activity contributes to improved mental health remain insufficiently understood. In particular, relatively limited attention has been paid to the unique role of organized group sports participation during adolescence.

For the purposes of this study, group sports participation refers to regular involvement in organized or semi-organized physical activities undertaken with other participants and characterized by repeated interpersonal contact, shared rules or goals, and some degree of coordinated participation (White-Gosselin et al., 2024; Yang et al., 2022). This conceptual definition distinguishes the social-organizational context of group sport from the dose of activity performed. PARS-3 was therefore used to operationalize the dose component exercise intensity, frequency, and duration reported within the study's group-sport frame, rather than to measure team interdependence, coaching structure, or social climate. Its brief format, established scoring procedure, and established use in Chinese samples supported its selection for a classroom-based adolescent survey (Liang and Liu, 1994); however, its inability to independently verify activity type remains an important limitation.

Compared with individual forms of exercise, organized group sports provide adolescents with opportunities for interpersonal interaction, emotional exchange, collective goal pursuit, and social integration (White-Gosselin et al., 2024). Participation in team-based sports contexts allows adolescents to experience peer affiliation, cooperation, and social recognition, all of which may contribute to psychological adaptation. Longitudinal evidence has suggested that positive social experiences within organized sports are associated with lower depressive symptoms and greater emotional well-being over time (Easterlin et al., 2019; Gómez-Baya et al., 2020). Moreover, adolescents who maintain stable engagement in group sports tend to exhibit more favorable psychosocial adjustment than their less active peers (Brière et al., 2018).

The Chinese context is theoretically relevant rather than merely a matter of sampling convenience. Chinese adolescents' daily routines are strongly organized around school, and academic pressure, parental expectations, and peer evaluation are prominent correlates of distress (Wang et al., 2020; Chyu and Chen, 2022; Zhou et al., 2024). Within this context, group sport may offer a recurring setting for competence feedback, peer affiliation, and collective participation outside exclusively academic evaluation. At the same time, access to and the meaning of group sport may vary with school resources, examination demands, family attitudes, and regional socioeconomic conditions. Studying a Chinese sample therefore allows the proposed self-esteem and perceived-social-support pathways to be examined in a context where academic and relational processes may be especially salient, while requiring caution when extending the findings to other educational or cultural settings.

Existing research has increasingly emphasized the importance of psychological and social resources in explaining the relationship between physical activity and mental health (Khan and Subhan, 2023). Among these factors, self-esteem and perceived social support have received considerable empirical attention. Self-esteem refers to an individual's overall evaluation of self-worth and personal value, whereas perceived social support reflects the extent to which individuals believe they receive emotional and instrumental support from significant others. Both variables have consistently been identified as protective factors against psychological distress during adolescence.

From a theoretical perspective, Social Cognitive Theory provides an important framework for understanding the association between physical activity and self-esteem (Bandura, 2001). According to Bandura's theory, repeated mastery experiences and positive performance feedback contribute to the development of favorable self-perceptions. Adolescents participating in organized group sports frequently encounter opportunities to develop athletic competence, achieve collective goals, and receive encouragement from peers and coaches. These experiences may strengthen adolescents' perceptions of competence and personal efficacy, thereby promoting higher levels of self-esteem.

In addition, Self-Determination Theory offers a useful framework for understanding the relationship between group sports participation and social support. Self-Determination Theory posits that the fulfillment of basic psychological needs, particularly the need for relatedness, contributes substantially to psychological well-being and adaptive functioning (Deci and Ryan, 2012). Organized sports environments inherently involve social interaction, cooperation, and shared identity formation, thereby creating conditions that facilitate interpersonal connectedness and social integration (Yang et al., 2022). Through regular engagement in group sports, adolescents may perceive greater support from teammates, friends, teachers, and family members, which may subsequently buffer against psychological distress.

Taken together, Social Cognitive Theory and Self-Determination Theory provide complementary grounds for the proposed structural model rather than separate post hoc explanations. Repeated mastery, performance feedback, and competence experiences in group-sport settings may strengthen self-esteem, whereas repeated relatedness experiences, peer affiliation, and team inclusion may strengthen perceived social support. These psychological and social resources can develop concurrently and may each be associated with lower distress through distinct self-evaluative and interpersonal pathways. Because the theoretical framework did not specify a necessary temporal ordering between self-esteem and perceived social support, the two mediators were modeled in parallel rather than as a causal chain. This structure represents a theory-guided hypothesis, not proof of causal direction; consequently, feasible competing explanations were examined within the constraints of the available measurement schedule.

Empirical findings have provided preliminary support for these theoretical assumptions. Previous studies involving adolescent samples have demonstrated that self-esteem partially mediates the relationship between physical activity and depressive symptoms (McPhie and Rawana, 2012). Similarly, perceived social support has also been identified as an important mediator linking physical activity with psychological well-being, life satisfaction, and emotional adjustment (Liao et al., 2023). However, most previous studies have examined self-esteem and social support independently or within cross-sectional designs, making it difficult to determine their unique prospective contributions within a single longitudinal framework.

Several limitations within the existing literature therefore warrant further investigation. First, although both self-esteem and social support have been identified as important explanatory mechanisms, relatively few studies have simultaneously examined these variables as parallel mediators in the relationship between group sports participation and adolescent mental health. Examining both mediators within the same model may clarify whether psychological and social resources independently contribute to the beneficial effects of physical activity. Second, the predominance of cross-sectional research designs limits the ability to establish temporal precedence and infer prospective associations among variables. Third, many previous longitudinal studies have failed to adequately control for baseline levels of mediators and mental health outcomes, thereby limiting the precision of estimated longitudinal effects.

Longitudinal mediation also requires careful attention to measurement timing and model specification. The validity of an indirect-effect estimate depends on whether the measurement lags correspond to the speed of the hypothesized processes and whether prior levels of the mediator and outcome are considered (Cole and Maxwell, 2003; Maxwell and Cole, 2007). In addition, conventional cross-lagged coefficients can combine stable between-person differences with within-person fluctuations (Hamaker et al., 2015). The present design was therefore treated as a temporally ordered, baseline-adjusted prospective mediation study rather than as a complete within-person causal panel.

To address these limitations, the present study employed a three-wave longitudinal design across one academic year. At T1, group-sport-related physical activity, self-esteem, perceived social support, and psychological distress were assessed. Self-esteem and perceived social support were reassessed at T2, and perceived social support and psychological distress were assessed at T3. The primary model focused on the temporally ordered pathway from T1 physical activity to T2 self-esteem and perceived social support and then to T3 psychological distress, while controlling for baseline levels of the mediators and psychological distress where available. This asymmetric measurement schedule establishes temporal ordering for the focal pathway but does not constitute a complete three-wave panel for every construct.

The measurement intervals were selected to align with the school calendar and the hypothesized pace of the processes. The approximately 4–6-month T1-to-T2 interval spanned most of an academic semester, allowing repeated sport-related experiences to accumulate before reassessing relatively stable self-evaluative and social resources. The shorter 1–2-month T2-to-T3 interval was intended to capture a more proximal association between these resources and psychological distress, consistent with the K6 reference period of the past month. There is no universally established optimal lag for changes in adolescent self-esteem or perceived social support; optimal timing depends on construct stability and the speed of the underlying process (Dormann and Griffin, 2015). Accordingly, the selected intervals were theory-informed and pragmatic, but they should not be assumed to be empirically optimal.

Based on the theoretical framework and previous empirical evidence, the present study proposed the following hypotheses:

1. H1: Higher group-sport-related physical activity at T1 will be prospectively associated with lower psychological distress at T3 after controlling for baseline psychological distress and available demographic variables.
2. H2: Self-esteem at T2 will account for a significant indirect association between group-sport-related physical activity at T1 and psychological distress at T3.
3. H3: Perceived social support at T2 will account for a significant indirect association between group-sport-related physical activity at T1 and psychological distress at T3.
4. H4: Self-esteem and perceived social support will show unique parallel indirect associations linking group-sport-related physical activity with psychological distress.

By examining these hypotheses using a temporally ordered longitudinal design, the present study seeks to provide evidence regarding prospective psychological and social pathways linking group-sport-related physical activity with adolescent mental health. The findings may inform school-based physical education and mental health promotion, while remaining subject to the sampling, measurement, and causal-inference boundaries described below.

## 2 Materials and methods

### 2.1 Participants

The present study employed a three-wave longitudinal design involving adolescents recruited from regular middle and high schools in Wenzhou, China. Prior to data collection, an a priori sample size estimation was conducted based on recommendations for longitudinal mediation models using structural equation modeling. Considering the anticipated small-to-moderate indirect effects reported in previous studies examining physical activity, psychosocial resources, and adolescent mental health, a minimum sample size of approximately 500 participants was considered necessary to achieve adequate statistical power (power = 0.80, α = 0.05) after accounting for potential attrition across waves.

Participants were recruited using convenience cluster sampling from four public schools, including two middle schools and two high schools. Eligibility criteria included: (a) being between 12 and 18 years of age; (b) current enrollment as a full-time student; (c) ability to complete self-report questionnaires independently; and (d) provision of informed consent from both students and their parents or legal guardians. Adolescents with severe cognitive impairments or physical conditions that prevented participation in regular physical activity were excluded. Because schools were selected on the basis of accessibility rather than probability sampling, the sample was not intended to be statistically representative of all adolescents in Wenzhou or China.

At baseline (T1), 677 valid questionnaires were collected. After excluding participants with substantial missing data, the final baseline sample consisted of 622 adolescents. Across the three measurement waves, 545 participants completed all assessments and were included in the final longitudinal analyses, corresponding to a retention rate of 87.6%. The final sample included 276 boys (50.6%) and 269 girls (49.4%), with a mean age of 14.9 years (SD = 1.6).

Attrition analyses were conducted to compare participants who completed all three waves with those who withdrew during the study period. Independent-samples *t*-tests and chi-square analyses indicated no significant differences in baseline demographic variables, PARS-3 physical activity scores, self-esteem, perceived social support, or psychological distress between completers and non-completers (all *p* > 0.05), suggesting that attrition did not systematically differentiate the two groups on the measured baseline characteristics.

### 2.2 Procedure

Data collection was conducted from October 2025 to April 2026. Ethical approval for the study was obtained from the Institutional Review Board of Wenzhou University. Permission to conduct the study was additionally obtained from participating schools and local educational authorities.

Prior to data collection, parents and students received detailed information regarding the purpose of the study, confidentiality procedures, and the voluntary nature of participation. Written informed consent was obtained from parents or legal guardians, while participating students provided assent before questionnaire administration.

At Time 1 (T1; October 2025), participants completed measures assessing demographic characteristics, group-sport-related physical activity dose, self-esteem, perceived social support, and psychological distress during classroom sessions supervised by trained research assistants. Participants were informed that their responses would remain anonymous and confidential and that there were no correct or incorrect answers. Completion of the questionnaire package required approximately 25 min.

The second wave of data collection (T2) was conducted approximately 4–6 months after baseline (March 2026), spanning most of the school semester so that repeated sport-related experiences could plausibly accumulate before self-esteem and perceived social support were reassessed. The third wave (T3) was conducted approximately 1–2 months after T2 (April 2026), when psychological distress was reassessed to capture a more proximal outcome period consistent with the K6 past-month reference frame. The unequal spacing also reflected the participating schools' academic calendar. Because no single optimal lag has been established for these developmental processes, the intervals should be regarded as theory-informed and pragmatic rather than definitive.

To maximize retention across measurement waves, several procedures were implemented. Class teachers assisted in reminding students about follow-up assessments, and small stationery gifts were provided following each wave of participation. Participants were informed that they could withdraw from the study at any time without penalty.

### 2.3 Instruments

#### 2.3.1 Group-sport-related physical activity (T1)

Group-sport-related physical activity dose was assessed using the Physical Activity Rating Scale-3 (PARS-3), a general physical activity instrument widely used among Chinese adolescents (Liang and Liu, 1994). The scale includes three items assessing exercise intensity, frequency, and duration. Exercise intensity is rated from 1 (low intensity) to 5 (high intensity), exercise frequency from 1 (less than once per week) to 5 (almost every day), and exercise duration from 1 (less than 30 min) to 5 (more than 90 min). PARS-3 does not contain an item that independently classifies an activity as group/team based versus individual. In the present study, it was used to quantify exercise dose within the group-sport frame of the survey; consequently, the resulting score should be interpreted as the amount of physical activity reported in relation to group-sport participation, not as a validated measure of team structure, organizational characteristics, or the exclusivity of group-sport involvement. Following established scoring procedures, the activity score was calculated by multiplying intensity, frequency, and duration scores, with higher scores indicating a greater reported exercise dose. In the present study, Cronbach's α coefficient was 0.941. PARS-3 was selected because the focal operational variable was the dose of activity undertaken within a group-sport frame, and its brevity made it feasible within a multi-measure classroom survey. It was considered appropriate for quantifying the intensity-frequency-duration dimension of participation, but not for classifying the social or organizational form of the activity; this distinction was maintained in the terminology and interpretation of the analyses.

#### 2.3.2 Self-esteem (T1 and T2)

Self-esteem was measured using the Chinese version of the Rosenberg Self-Esteem Scale (RSES) (Rosenberg, 1965). The scale consists of 10 items assessing global self-worth and self-acceptance. Responses are rated on a 4-point Likert scale ranging from 1 (strongly disagree) to 4 (strongly agree). Negatively worded items were reverse coded prior to analysis, with higher scores reflecting higher levels of self-esteem. The Chinese version of the RSES has demonstrated satisfactory psychometric properties in adolescent samples. In the current study, Cronbach's α coefficients were 0.972 at T1 and 0.976 at T2.

#### 2.3.3 Social support (T1, T2, T3)

Perceived social support was assessed using the Chinese version of the Multidimensional Scale of Perceived Social Support (MSPSS) (Zimet et al., 1988). The MSPSS comprises 12 items evaluating perceived support from family, friends, and significant others. Responses are recorded on a 7-point Likert scale ranging from 1 (very strongly disagree) to 7 (very strongly agree), with higher scores indicating greater perceived social support. The Chinese version of the MSPSS has shown strong reliability and construct validity in adolescent populations. In the present study, Cronbach's α coefficients were 0.982 at T1, 0.983 at T2 and 0.985 at T3.

#### 2.3.4 Psychological health (T1 and T3)

Psychological distress was assessed using the Kessler Psychological Distress Scale (K6) (Kessler et al., 2002). The K6 includes six items measuring the frequency of nonspecific psychological distress symptoms experienced during the past month, including nervousness, hopelessness, restlessness, depressive mood, effortfulness, and feelings of worthlessness. Responses are rated on a 5-point Likert scale ranging from 1 (none of the time) to 5 (all of the time). Higher scores indicate higher levels of psychological distress. The Chinese version of the K6 has demonstrated good reliability and validity among adolescents. In the present study, Cronbach's α coefficients were 0.960 at T1 and 0.967 at T3.

### 2.4 . Data analysis

All statistical analyses were conducted using SPSS version 26.0 and AMOS version 24.0. The analytic procedures were organized in accordance with the sequence of the results reported in the present study. The analyses were designed to estimate prospective associations and indirect effects; they were not treated as estimates of definitive causal effects.

First, preliminary analyses were conducted to describe the main study variables. Means, standard deviations, skewness, kurtosis, minimum values, and maximum values were calculated for T1 PARS-3 physical activity scores, self-esteem at T1 and T2, perceived social support at T1, T2, and T3, and psychological distress at T1 and T3. These analyses were used to evaluate the central tendency, variability, and distributional characteristics of the variables.

Second, internal consistency reliability was examined for each scale at each available measurement wave. Cronbach's α coefficients were calculated for the PARS-3, RSES, MSPSS, and K6. Reliability coefficients greater than.70 were considered acceptable.

Third, Pearson correlation analyses were performed to examine the bivariate associations among T1 PARS-3 physical activity scores, self-esteem, perceived social support, and psychological distress across the available measurement waves.

Fourth, missing data and attrition were examined. Missing data patterns were evaluated using Little's Missing Completely at Random (MCAR) test. Full information maximum likelihood estimation was used to handle missing data in the longitudinal analyses. Attrition analyses were conducted to compare participants who completed all three waves with those who did not complete the full study.

Fifth, the measurement model was evaluated using confirmatory factor analysis. Model fit was assessed using the chi-square to degrees of freedom ratio (χ^2^/df), Comparative Fit Index (CFI), Tucker–Lewis Index (TLI), Root Mean Square Error of Approximation (RMSEA), and Standardized Root Mean Square Residual (SRMR).

Sixth, longitudinal measurement invariance was examined across measurement waves. Configural, metric, and scalar invariance models were estimated sequentially. Changes in CFI and RMSEA were used to evaluate whether increasingly constrained models remained acceptable.

Seventh, the hypothesized temporally ordered parallel mediation model was tested using structural equation modeling. Group-sport-related physical activity at T1 was specified as the independent variable, self-esteem and perceived social support at T2 were specified as parallel mediators, and psychological distress at T3 was specified as the outcome. Prior levels of self-esteem, perceived social support, and psychological distress were included where available, together with the available demographic covariates. Socioeconomic status, academic stress, and physical health were not available as covariates. Because physical activity was assessed only at T1, psychological distress was not assessed at T2, and self-esteem was not assessed at T3, the model represents an asymmetric or half-longitudinal mediation design and cannot estimate all reciprocal or change-to-change pathways.

Eighth, indirect effects were tested using bias-corrected bootstrap procedures based on 5,000 resamples. The indirect effect through self-esteem, the indirect effect through perceived social support, the indirect effect, the direct effect, and the effect were estimated. Indirect effects were considered statistically significant when the 95% confidence intervals did not include zero.

Ninth, the relative strength of the two mediating pathways was compared by examining the difference between the indirect effect through self-esteem and the indirect effect through perceived social support.

Tenth, sensitivity analyses compared the hypothesized parallel mediation model with a feasible reversed psychosocial-direction model in which baseline psychological distress, rather than T1 PARS-3, was specified as the principal antecedent of T2 self-esteem and perceived social support, while retaining autoregressive controls and the paths from T2 psychosocial resources to T3 psychological distress. Model comparison was based on the Akaike Information Criterion (AIC) and Bayesian Information Criterion (BIC), with lower values indicating better relative fit. Because later sports participation was not measured, a fully reversed model in which psychological distress predicts subsequent sports participation could not be estimated. Likewise, the asymmetric schedule did not permit a complete comparison of all sequential mediation alternatives. The sensitivity analysis therefore evaluates one plausible competing structure rather than establishing exhaustive causal superiority.

An RI-CLPM was not estimated. This model requires repeated observations of each focal construct across sufficient waves to separate stable between-person differences from within-person deviations. In the present study, PARS-3 was available only at T1, self-esteem at T1 and T2, and psychological distress at T1 and T3; therefore, an RI-CLPM for the focal variables was not identifiable. The reported lagged coefficients were consequently interpreted as exploratory conventional prospective associations at the between-person level, not as evidence that within-person changes in one construct caused later within-person changes in another (Hamaker et al., 2015).

Finally, common method bias was evaluated using Harman's single-factor test. The percentage of variance explained by the first unrotated factor was examined, with values below 40% interpreted as indicating that common method bias was unlikely to substantially influence the results.

## 3 Results

### 3.1 Sample description

Descriptive statistics for the primary study variables are presented in Table 1. At T1, the mean PARS-3 physical activity score was 41.89 (SD = 42.74), with scores ranging from 1 to 125. The mean RSES scores were 25.02 (SD = 10.02) at T1 and 24.90 (SD = 10.12) at T2. Mean perceived social support scores were 48.25 (SD = 22.14) at T1, 48.04 (SD = 21.94) at T2, and 48.11 (SD = 22.14) at T3. Psychological distress scores measured by the K6 averaged 17.81 (SD = 7.80) at T1 and 18.02 (SD = 7.88) at T3. Across all variables, skewness values ranged from −0.07 to 0.82, and kurtosis values ranged from −1.32 to −0.74, indicating no substantial deviations from normality.

**Table 1:** Descriptive statistics of study variables.

Variables	Mean	SD	Skewness	Kurtosis	Min	Max
PARS T1	41.89	42.74	−0.82	0.74	1	125
RSES T1	25.02	10.02	−0.02	−1.3	10	40
RSES T2	24.9	10.12	−0.01	−1.32	10	40
MSPSS T1	48.25	22.14	−0.07	−1.2	12	84
MSPSS T2	48.04	21.94	−0.01	−1.17	12	84
MSPSS T3	48.11	22.14	−0.03	−1.13	12	84
K6 T1	17.81	7.8	0.03	−1.28	6	30
K6 T3	18.02	7.88	−0.0	−1.26	6	30

### 3.2 Reliability analyses

Cronbach's α coefficients for all study measures are presented in Table 2. The PARS-3 demonstrated high internal consistency at T1 (α = 0.941). The RSES showed excellent reliability at both T1 (α = 0.972) and T2 (α = 0.976). The MSPSS demonstrated consistently high internal consistency across all three measurement waves, with Cronbach's α coefficients of 0.982 at T1,0.983 at T2, and 0.985 at T3. The K6 also exhibited excellent reliability, with α coefficients of 0.960 at T1 and 0.967 at T3. Overall, all scales demonstrated satisfactory internal consistency across measurement occasions.

**Table 2:** Reliability analyses.

Variables	T1 α	T2 α	T3 α
PARS-3	0.941		
RSES	0.972	0.976	
MSPSS	0.982	0.983	0.985
K6	0.960		0.967

### 3.3 Correlation analyses

**Figure 1:**
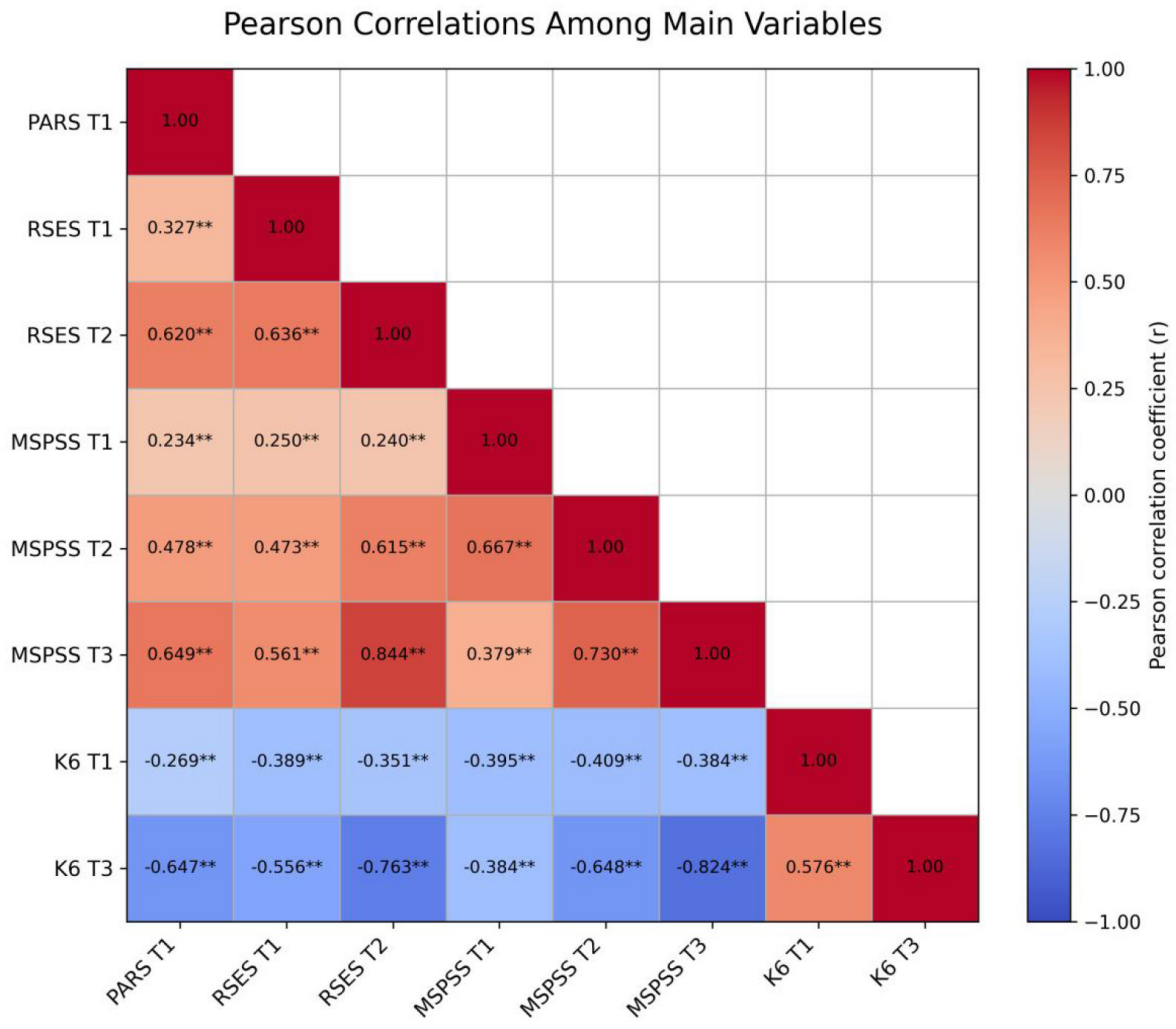
Heatmap of correlations among key variables. **p < 0.01.

Pearson correlation coefficients among the primary study variables are presented in Table 3 and Figure 1. T1 PARS-3 physical activity scores were positively correlated with self-esteem at T1 (*r* = 0.327, *p* < 0.01) and T2 (*r* = 0.620, *p* < 0.01), and with perceived social support at T1 (*r* = 0.234, *p* < 0.01), T2 (*r* = 0.478, *p* < 0.01), and T3 (*r* = 0.649, *p* < 0.01). In contrast, T1 PARS-3 scores were negatively correlated with psychological distress at T1 (*r* = −0.269, *p* < 0.01) and T3 (*r* = −0.647, *p* < 0.01). Self-esteem at T2 was positively associated with perceived social support at T2 (*r* = 0.615, *p* < 0.01) and T3 (*r* = 0.844, *p* < 0.01), and negatively associated with psychological distress at T3 (*r* = −0.763, *p* < 0.01). Perceived social support at T2 and T3 was also negatively correlated with psychological distress at T3 (*r* = −0.648 and *r* = −0.824, respectively, both *p* < 0.01). Psychological distress demonstrated moderate temporal stability, with T1 distress positively associated with T3 distress (*r* = 0.576, *p* < 0.01). These bivariate correlations are descriptive and do not establish temporal or causal direction.

**Table 3:** Pearson correlations among main variables.

Variables	PARS T1	RSES T1	RSES T2	MSPSS T1	MSPSS T2	MSPSS T3	K6 T1	K6 T3
PARS T1	1							
RSES T1	0.327^**^	1						
RSES T2	0.620^**^	0.636^**^	1					
MSPSS T1	0.234^**^	0.250^**^	0.240^**^	1				
MSPSS T2	0.478^**^	0.473^**^	0.615^**^	0.667^**^	1			
MSPSS T3	0.649^**^	0.561^**^	0.844^**^	0.379^**^	0.730^**^	1		
K6 T1	−0.269^**^	−0.389^**^	−0.351^**^	−0.395^**^	−0.409^**^	−0.384^**^	1	
K6 T3	−0.647^**^	−0.556^**^	−0.763^**^	−0.384^**^	−0.648^**^	−0.824^**^	0.576^**^	1

^**^p < 0.01.

### 3.4 Missing data and attrition analyses

Missing data patterns were examined using Little's Missing Completely at Random (MCAR) test. As shown in Table 4, the results indicated that the missing data pattern did not significantly deviate from randomness, χ^2^_(74)_ = 86.41, *p* = 0.154, suggesting that the assumption of MCAR/MAR was acceptable. Accordingly, full information maximum likelihood (FIML) estimation was applied in subsequent longitudinal analyses to handle incomplete data.

**Table 4:** Missing data and attrition analyses.

Indicator	Result
Little's MCAR test	χ^2^ = 86.41, df = 74, *p* = 0.154
Missing data handling	FIML
Conclusion	Missingness approximated MCAR/MAR assumptions

### 3.5 Confirmatory factor analysis

The measurement model demonstrated satisfactory fit to the data. As shown in Table 5, the chi-square to degrees of freedom ratio was 1.96, the Comparative Fit Index (CFI) was 0.962, and the Tucker–Lewis Index (TLI) was 0.951. In addition, the Root Mean Square Error of Approximation (RMSEA) was 0.042 and the Standardized Root Mean Square Residual (SRMR) was 0.041. Collectively, these indices indicated acceptable model fit.

**Table 5:** Measurement model fit indices.

Index	Value
χ^2^/df	1.96
CFI	0.962
TLI	0.951
RMSEA	0.042
SRMR	0.041

### 3.6 Longitudinal measurement invariance

Longitudinal measurement invariance analyses are presented in Table 6. The configural invariance model demonstrated acceptable fit to the data (CFI = 0.958, RMSEA = 0.044). Constraining factor loadings to equality across time resulted in minimal changes in model fit (ΔCFI = 0.004, ΔRMSEA = 0.002), supporting metric invariance. Similarly, the scalar invariance model also demonstrated acceptable fit, with small changes in fit indices relative to the metric model (ΔCFI = 0.005, ΔRMSEA = 0.003). These findings supported longitudinal measurement invariance across measurement waves.

**Table 6:** Longitudinal measurement invariance.

Model	CFI	RMSEA	ΔCFI	ΔRMSEA
Configural	0.958	0.044	–	–
Metric	0.954	0.046	0.004	0.002
Scalar	0.949	0.049	0.005	0.003

### 3.7 Structural model testing

The standardized structural path coefficients are presented in Table 7. Higher T1 PARS-3 physical activity scores were associated with lower psychological distress at T3 (β = −0.528, SE = 0.029, *p* < 0.001), higher self-esteem at T2 (β = 0.462, SE = 0.029, *p* < 0.001), and greater perceived social support at T2 (β = 0.342, SE = 0.030, *p* < 0.001). Higher T2 self-esteem (β = −0.416, SE = 0.032, *p* < 0.001) and perceived social support (β = −0.157, SE = 0.029, *p* < 0.001) were associated with lower T3 psychological distress. T1 psychological distress was positively associated with T3 psychological distress (β = 0.303, SE = 0.024, *p* < 0.001), indicating temporal stability. These coefficients represent adjusted prospective associations rather than experimentally identified causal effects.

**Table 7:** Structural path coefficients.

Path	Std β	SE	*P*
PARS T1 → K6 T3	−0.528	0.029	< 0.001
PARS T1 → RSES T2	0.462	0.029	< 0.001
PARS T1 → MSPSS T2	0.342	0.03	< 0.001
RSES T2 → K6 T3	−0.416	0.032	< 0.001
MSPSS T2 → K6 T3	−0.157	0.029	< 0.001
K6 T1 → K6 T3	0.303	0.024	< 0.001

**Figure 2:**
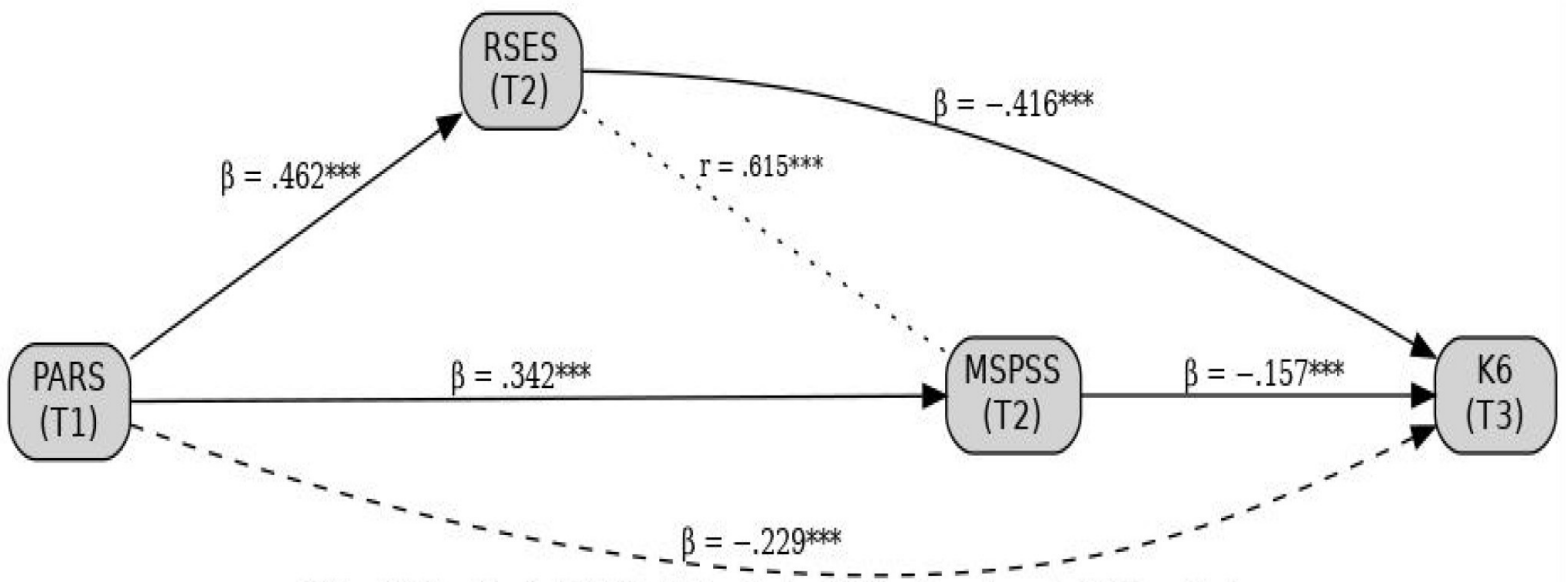
Path diagram of the parallel mediation model. PARS, Physical Activity Rating Scale; RSES, Rosenberg Self-Esteem Scale; MSPSS, Multidimensional Scale of Perceived Social Support; K6, Kessler Psychological Distress Scale. Standardized coefficients are shown. ***p < 0.001.

The parallel mediation estimates are presented in Table 8 and Figure 2. The estimated indirect association through self-esteem was significant (β = −0.192, 95% CI [−0.235, −0.151], *p* < 0.001), as was the estimated indirect association through perceived social support (β = −0.054, 95% CI [−0.081, −0.029], *p* < 0.001). The combined indirect association was significant (β = −0.246, 95% CI [−0.309, −0.189], *p* < 0.001). The adjusted direct association between T1 PARS-3 scores and T3 psychological distress remained significant after including the mediators (β = −0.229, 95% CI [−0.301, −0.157], *p* < 0.001), and the total association was also significant (β = −0.475, 95% CI [−0.542, −0.411], *p* < 0.001). The terms direct, indirect, and total effect in Table 8 refer to the conventional statistical decomposition used in structural equation modeling and should not be interpreted as experimentally identified causal effects.

**Table 8:** Parallel mediation effects.

Effect	Standardized β	95% CI	*p*
Via RSES	−0.192	[−0.235, −0.151]	< 0.001
Via social support	−0.054	[−0.081, −0.029]	< 0.001
Total indirect effect	−0.246	[−0.309, −0.189]	< 0.001
Direct effect	−0.229	[−0.301, −0.157]	< 0.001
Total effect	−0.475	[−0.542, −0.411]	< 0.001

The comparative strengths of the two indirect pathways are presented in Table 9. The indirect effect through RSES (β = 0.192) was larger than the indirect effect through perceived social support (β = 0.054). The contrast between the two mediation effects was 0.139.

**Table 9:** Comparative strength of mediators.

Comparison	Effect size
RSES indirect effect	−0.192
Social support indirect effect	−0.054
Difference (contrast)	0.139

### 3.8 Exploratory autoregressive and lagged path analyses

**Figure 3:**
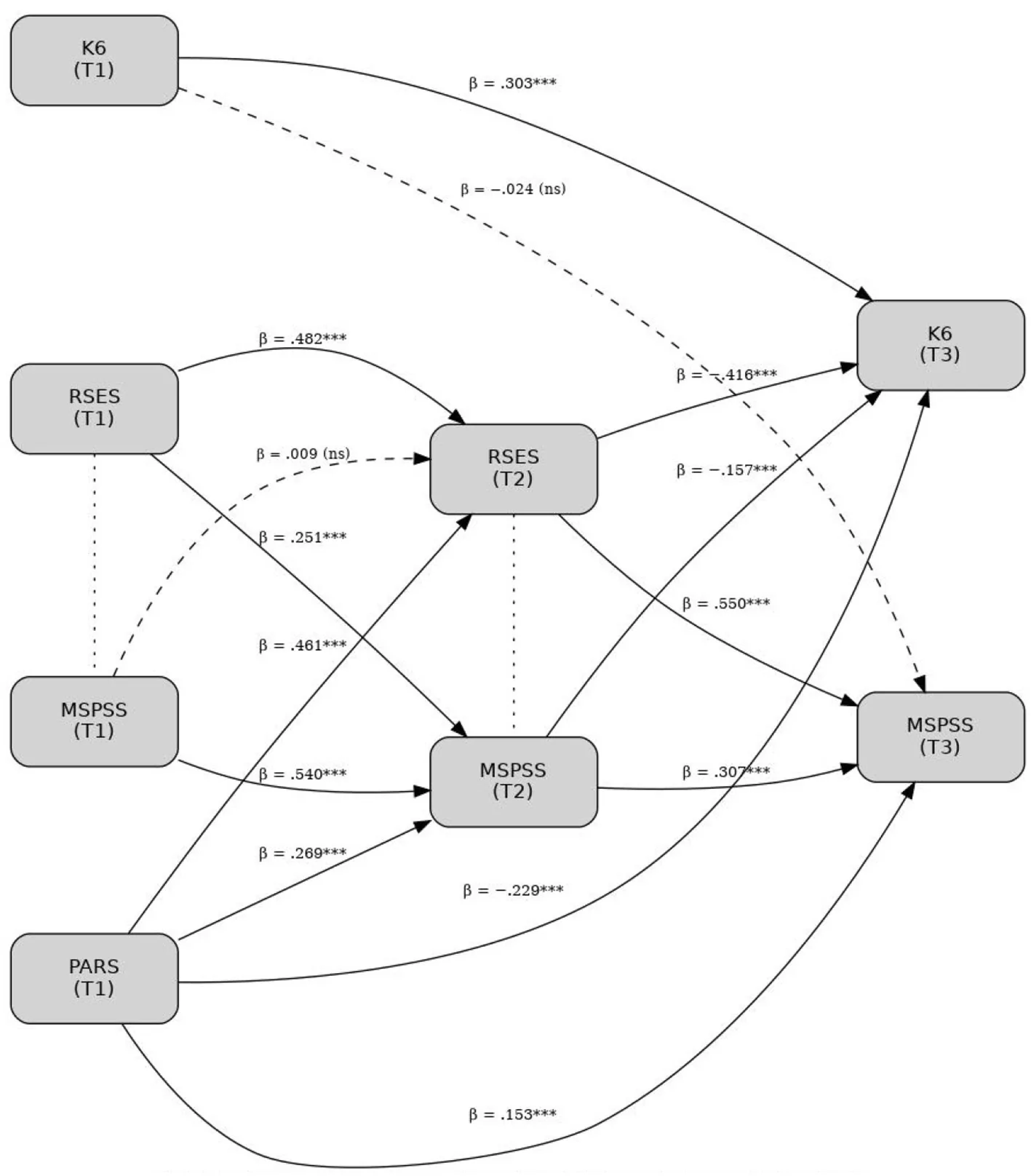
Exploratory lagged path model coefficient diagram. Standardized coefficients are presented. Dashed lines indicate non-significant paths. ***p < 0.001; ns, non-significant. PARS, Physical Activity Rating Scale; RSES Rosenberg Self-Esteem Scale; MSPSS, Multidimensional Scale of Perceived Social Support; K6, Kessler Psychological Distress Scale.

The key exploratory autoregressive and lagged path coefficients are presented in Table 10 and Figure 3. Self-esteem at T1 was positively associated with self-esteem at T2 (β = 0.482, SE = 0.030, *p* < 0.001), whereas perceived social support at T1 was not significantly associated with self-esteem at T2 (β = 0.009, SE = 0.029, *p* = 0.745). T1 PARS-3 scores were positively associated with self-esteem at T2 (β = 0.461, SE = 0.030, *p* < 0.001). Perceived social support at T1 was positively associated with perceived social support at T2 (β = 0.540, SE = 0.028, *p* < 0.001). In addition, self-esteem at T1 was positively associated with perceived social support at T2 (β = 0.251, SE = 0.029, *p* < 0.001), and T1 PARS-3 scores were also positively associated with perceived social support at T2 (β = 0.269, SE = 0.029, *p* < 0.001). Psychological distress at T1 was positively associated with psychological distress at T3 (β = 0.303, SE = 0.024, *p* < 0.001). Self-esteem at T2 (β = −0.416, SE = 0.032, *p* < 0.001), perceived social support at T2 (β = −0.157, SE = 0.029, *p* < 0.001), and T1 PARS-3 scores (β = −0.229, SE = 0.028, *p* < 0.001) were negatively associated with psychological distress at T3. Perceived social support at T2 was positively associated with perceived social support at T3 (β = 0.307, SE = 0.026, *p* < 0.001), and self-esteem at T2 was positively associated with perceived social support at T3 (β = 0.550, SE = 0.028, *p* < 0.001). Baseline psychological distress was not significantly associated with perceived social support at T3 (β = −0.024, SE = 0.022, *p* = 0.275), whereas T1 PARS-3 scores were positively associated with perceived social support at T3 (β = 0.153, SE = 0.025, *p* < 0.001). The explained variance for the outcome models ranged from 59.7% to 79.9% (*R*^2^ = 0.597–0.799). Because stable between-person differences were not separated through random intercepts, these estimates should not be interpreted as within-person dynamic effects.

**Table 10:** Key exploratory autoregressive and lagged paths.

Variables	Predictor	Std_Beta	SE	*t*	*p*	95% CI lower	95% CI upper	*R* ^2^	Adj_R^2^
RSES T2	RSES T1	0.482	0.03	16.274	< 0.001	0.424	0.541	0.597	0.592
RSES T2	MSPSS T1	0.009	0.029	0.326	0.745	−0.047	0.066	0.597	0.592
RSEST2	PARST1	0.461	0.03	15.579	< 0.001	0.402	0.519	0.597	0.592
MSPSS T2	MSPSS T1	0.54	0.028	19.121	< 0.001	0.485	0.596	0.611	0.606
MSPSS T2	RSES T1	0.251	0.029	8.611	< 0.001	0.194	0.308	0.611	0.606
MSPSS T2	PARST1	0.269	0.029	9.268	< 0.001	0.212	0.326	0.611	0.606
K6 T3	K6 T1	0.303	0.024	12.541	< 0.001	0.256	0.351	0.746	0.742
K6 T3	RSES T2	−0.416	0.032	−13.2	< 0.001	−0.478	−0.354	0.746	0.742
K6 T3	MSPSS T2	−0.157	0.029	−5.426	< 0.001	−0.214	−0.1	0.746	0.742
K6 T3	PARST1	−0.229	0.028	−8.099	< 0.001	−0.285	−0.173	0.746	0.742
MSPSS T3	MSPSS T2	0.307	0.026	11.896	< 0.001	0.256	0.358	0.799	0.796
MSPSS T3	RSES T2	0.55	0.028	19.595	< 0.001	0.495	0.605	0.799	0.796
MSPSS T3	K6 T1	−0.024	0.022	−1.094	0.275	−0.066	0.019	0.799	0.796
MSPSS T3	PARST1	0.153	0.025	6.082	< 0.001	0.104	0.203	0.799	0.796

### 3.9 Sensitivity analyses

Sensitivity analyses are presented in Table 11. The hypothesized parallel mediation model demonstrated lower Akaike Information Criterion (AIC = 1091.34) and Bayesian Information Criterion (BIC = 1174.83) values than the tested reversed psychosocial-direction model (AIC = 1248.52; BIC = 1320.66). These information criteria indicate better relative fit for the hypothesized model among the two candidate structures that could be estimated. They do not establish causal superiority, because the alternative comparison was constrained by the asymmetric measurement schedule and did not include a later measure of sports participation.

**Table 11:** Sensitivity analyses.

Analysis	Result
Reversed psychosocial-direction model	AIC = 1248.52; BIC = 1320.66
Hypothesized parallel mediation model	AIC = 1091.34; BIC = 1174.83
Conclusion	Lower AIC/BIC for the hypothesized model among the tested candidates

### 3.10 Common method bias

The results of Harman's single-factor test are presented in Table 12. The first unrotated factor accounted for 29.4% of the total variance, which was below the commonly used threshold of 40%. This result suggests that a dominant single factor was not evident, although Harman's test is a limited diagnostic and does not eliminate all concerns associated with common-source self-report data.

**Table 12:** Common method bias test.

Indicator	Result
First unrotated factor variance explained	29.4%
Threshold	< 40%
Conclusion	No serious common method bias

All statistical estimates are calculated directly from the longitudinal analysis sample (*N* = 545). Standardized coefficients are reported. Bootstrap confidence intervals were estimated using 5,000 resamples.

## 4 Discussion

The present study examined prospective associations among group-sport-related physical activity, self-esteem, perceived social support, and psychological distress among adolescents using a three-wave, asymmetrically measured design. The study tested whether self-esteem and perceived social support at T2 showed parallel indirect associations between T1 PARS-3 scores and T3 psychological distress, while accounting for prior levels where available. Exploratory autoregressive and lagged paths were also examined. Overall, the results were consistent with the hypothesized model, but the convenience sample, incomplete panel, unmeasured confounding, and inability to separate within-person from between-person variation require cautious interpretation.

### 4.1 Group-sport-related physical activity and psychological distress

Consistent with the first hypothesis, higher T1 PARS-3 scores were prospectively associated with lower psychological distress at T3 after adjustment for baseline psychological distress and the available demographic covariates. This temporal pattern describes an inverse prospective association between sport-related physical activity and distress; it does not establish that change in group-sport participation produced change in psychological distress.

The present findings are consistent with previous research demonstrating an inverse association between physical activity and adolescent mental health problems. Earlier studies have reported that regular participation in sports and physical activity is associated with reduced depressive symptoms, lower anxiety, and improved emotional wellbeing among adolescents (McMahon et al., 2017). The current study extends this literature by examining PARS-3 exercise dose within a group-sport research frame and by adjusting for baseline psychological distress; however, PARS-3 itself does not distinguish team-based from individual exercise.

In this study, PARS-3 was appropriate for estimating the intensity-frequency-duration dimension of activity reported under a group-sport frame, which was the exposure dose used in the structural model. It was not appropriate for determining whether the same dose occurred in a formal team, an informal peer group, or an individual setting. Accordingly, the findings should be understood as associations involving group-sport-related physical activity dose, not as evidence that the social structure of group sport uniquely accounts for the observed pattern.

Several processes are compatible with the observed association between sport-related physical activity and psychological adjustment. Compared with solitary exercise, organized group sports can involve social interaction, collective goal pursuit, peer cooperation, and shared emotional experiences (Andersen et al., 2019; White-Gosselin et al., 2024). Such contexts may provide opportunities for emotional regulation, adaptive coping, and social connection (Martín-Rodríguez et al., 2024). Regular physical activity may also coincide with stress reduction, better sleep, and neurobiological regulation. These explanations are theoretically plausible interpretations, however, and were not directly tested as mechanisms in the present observational design.

The adjusted direct association between T1 PARS-3 scores and T3 psychological distress remained significant after self-esteem and perceived social support were included. This residual statistical association indicates that the two measured indirect pathways did not account for the entire prospective relationship; it does not establish the existence of additional causal mechanisms. Future complete-panel studies could examine other candidate variables, such as resilience, emotion regulation, athletic identity, school connectedness, sleep, or physical fitness.

### 4.2 The prospective indirect association through self-esteem

The estimated indirect association through self-esteem supported the second hypothesis. Adolescents with higher T1 PARS-3 scores tended to report higher self-esteem at T2, which was in turn associated with lower psychological distress at T3. This pattern is consistent with Social Cognitive Theory, which emphasizes mastery experiences, competence development, and positive feedback in shaping self-perceptions. Sport settings are often characterized by opportunities for skill acquisition, achievement, and social recognition (Barber et al., 2005; Logan et al., 2019). Such experiences are conceptually relevant to competence and self-worth, although the present data did not assess them directly or establish that they accounted for the observed association.

The current findings also align with research identifying self-esteem as a psychological resource associated with both physical activity and mental health outcomes (Li and Hao, 2025). Adolescents with higher self-esteem generally report lower depression, anxiety, and emotional distress, and positive self-evaluations may be relevant to coping with interpersonal and emotional stressors (Fiorilli et al., 2019). Self-esteem may be particularly salient during adolescence, a developmental period characterized by heightened self-awareness, identity exploration, and sensitivity to social evaluation. The present data support a prospective indirect association but do not demonstrate that sport-induced change in self-esteem caused later change in distress.

The estimated indirect association through self-esteem was larger than that through perceived social support. This comparison indicates that self-esteem carried more of the model-based statistical association in this sample; it should not be interpreted as proof that self-esteem is the dominant causal mechanism. Replication with complete repeated measures and experimental or quasi-experimental designs is needed before stronger process claims are made.

### 4.3 The prospective indirect association through perceived social support

The estimated indirect association through perceived social support also supported the third hypothesis. Adolescents with higher T1 PARS-3 scores tended to report greater perceived social support at T2, which was subsequently associated with lower psychological distress at T3. This pattern is compatible with Self-Determination Theory, particularly the role of relatedness satisfaction in psychological functioning and wellbeing. Nevertheless, because PARS-3 does not directly measure team structure or social interaction, the social pathway should be interpreted as a prospective association requiring replication with a group-sport-specific exposure measure.

An inverse association between perceived social support and adolescent psychological distress has been consistently reported. Supportive relationships may provide emotional reassurance, stress-buffering resources, and opportunities for adaptive coping (Camara et al., 2017). In the present model, perceived social support at T2 accounted for a significant prospective indirect association between T1 PARS-3 scores and T3 distress. This statistical mediation pattern is consistent with, but does not establish, a causal social-support pathway.

### 4.4 Comparison of the two prospective indirect associations

An important contribution of the present study was the simultaneous estimation of self-esteem and perceived social support as parallel mediators within the same temporally ordered model. The estimated indirect association through self-esteem was larger than the corresponding association through perceived social support.

The difference in magnitude describes the fitted model in this sample and does not establish that self-esteem is causally more important than perceived social support. Both variables supplied unique explanatory information, but their relative roles require replication with complete repeated measures and designs capable of stronger causal identification.

### 4.5 Exploratory lagged and longitudinal associations

The exploratory conventional lagged analyses indicated that self-esteem at T1 was associated with perceived social support at T2 after accounting for prior perceived social support, whereas T1 perceived social support was not significantly associated with T2 self-esteem after accounting for prior self-esteem. These results describe differences between adolescents with different prior levels of the constructs; they do not demonstrate a unidirectional within-person process.

At the between-person level, adolescents with more positive self-evaluations may interact more confidently, display greater interpersonal openness, or interpret social experiences more positively, which could contribute to higher perceived support. However, the conventional lagged model does not separate these possibilities from stable characteristics such as sociability, family environment, or general positive affect. Accordingly, the non-significant reverse path should not be interpreted as evidence that social support cannot influence self-esteem within individuals.

Both T2 psychosocial variables were prospectively associated with lower T3 distress, while T1 PARS-3 scores were associated with later perceived support. These estimates remain between-person prospective associations; neither the significant nor the non-significant paths establish the presence or absence of within-person change.

### 4.6 Sociocultural interpretation in the chinese context

The observed pattern should also be interpreted in relation to Chinese adolescents' educational and relational environment. Academic achievement, high-stakes examinations, parental expectations, and peer comparison can be salient sources of self-evaluation and distress (Wang et al., 2020; Chyu and Chen, 2022; Zhou et al., 2024). In this setting, group-sport participation may coincide with opportunities for competence recognition, non-academic identity, peer affiliation, and collective belonging. These possibilities are consistent with the self-esteem and perceived-support associations, but the study did not directly measure cultural values or sport-specific experiences.

At the same time, school timetables, examination preparation, family attitudes toward sport, and access to facilities or extracurricular programs may influence both who participates and what participation means. Relational norms emphasizing cooperation and group belonging may make perceived support salient for some adolescents, whereas performance-focused or highly selective sport environments may not provide the same experience. The results should therefore be viewed as context-dependent associations within the participating Chinese public schools rather than as a uniform cultural effect.

### 4.7 Theoretical implications

The present study contributes to the literature by establishing temporal ordering for a theory-guided prospective pathway from T1 physical activity to T2 psychosocial resources and T3 psychological distress. Temporal separation and baseline adjustment reduce some ambiguities of cross-sectional mediation, but they do not by themselves establish causality, especially when exposure, mediators, and outcome are not all measured at every wave.

Simultaneously estimating self-esteem and perceived social support provides a theory-informed description of competence-related and relational resources, and the observed pattern is compatible with Social Cognitive Theory and Self-Determination Theory. However, theoretical compatibility and temporal ordering do not confirm the proposed mechanisms. The lagged self-esteem-to-support association should be treated as a hypothesis for future complete-panel RI-CLPM or intervention research.

### 4.8 Practical implications

The findings may inform the design of school-based research but do not demonstrate intervention effectiveness. Inclusive group-sport and team-based physical-education settings may offer opportunities for physical activity, competence feedback, and social interaction; whether expanding such opportunities is associated with lower distress should be evaluated in intervention studies.

Future trials should specify the sport format, assess implementation fidelity and social climate, and examine whether changes in self-esteem or perceived support accompany changes in psychological distress. Potential adverse experiences, subgroup differences, and unequal access should also be evaluated.

### 4.9 Limitations and future directions

Several limitations should be acknowledged when interpreting the findings. First, all variables were assessed using self-report questionnaires, which may increase shared-method and response biases. Moreover, PARS-3 quantifies general exercise intensity, frequency, and duration but does not independently distinguish group sports from individual exercise or capture team interdependence, coaching, organizational structure, or social climate. The present findings therefore concern exercise dose interpreted within a group-sport research frame, and activity-type misclassification cannot be ruled out. Future studies should combine activity-specific logs, objective activity monitoring, and validated team- or group-sport participation measures.

Second, the convenience cluster sample was drawn from four public middle and high schools in Wenzhou. Because neither schools nor students were selected through probability sampling, the findings should not be generalized as population estimates for Wenzhou or China. The most defensible boundary of generalization is to enrolled adolescents aged 12–18 attending public schools in urban or urbanizing Chinese settings with broadly similar educational structures and cultural expectations. Generalization to rural regions, other provinces or countries, private or vocational schools, out-of-school youth, younger children, older emerging adults, or cultural contexts with different meanings of organized sport requires direct replication.

Regional variation within China may be especially important. Schools differ in the time allocated to physical education, availability of facilities and trained staff, academic competition, and access to extracurricular clubs, while family socioeconomic resources may shape the affordability and continuity of participation. The sampled Wenzhou public schools may therefore offer activity opportunities and support networks that differ from those in rural, inland, lower-resource, private, or vocational settings. Such differences could affect both PARS-3 scores and the strength of their associations with self-esteem, perceived support, and distress; multisite sampling and explicit measurement of school- and family-level socioeconomic factors are needed.

Third, the measurement schedule was asymmetric: physical activity was assessed only at T1, psychological distress was not assessed at T2, and self-esteem was not assessed at T3. Although the focal T1-to-T2-to-T3 sequence provides temporal ordering and includes baseline controls where available, it does not form a complete panel. Consequently, the study cannot determine whether changes in sports participation covary with changes in the mediators or outcome, cannot test all reciprocal pathways, and cannot rule out the possibility that T1 PARS-3 scores function partly as a proxy for a stable active, socially engaged, or health-oriented disposition.

Fourth, potentially important confounders—including socioeconomic status, academic stress, physical health, prior mental health treatment, family functioning, and school climate—were not measured or controlled. These factors may influence both physical activity and psychological distress, and their omission may bias direct and indirect associations. The results should therefore be described as adjusted prospective associations rather than causal effects. Future studies should prespecify a confounder set using a causal framework and collect these variables at each wave.

Fifth, an RI-CLPM could not be estimated because the focal constructs were not each assessed at three or more waves. The conventional lagged paths may therefore reflect stable between-person differences rather than within-person dynamics. In particular, the association from T1 self-esteem to T2 perceived social support cannot be interpreted as evidence that an individual increase in self-esteem produces a later within-person increase in perceived support. A complete panel with all constructs measured at every wave is needed to test this proposition more directly.

Sixth, the measurement intervals were unequal. The longer T1-to-T2 interval was intended to allow psychosocial resources to develop across a semester, whereas the shorter T2-to-T3 interval targeted a proximal distress outcome. Nevertheless, if the true effects unfold more rapidly, the longer lag may attenuate or obscure them; if they unfold more slowly, the shorter lag may capture only partial change. Unequal lags also limit direct comparison of coefficient magnitudes across intervals. Future work should use more frequent and equally spaced assessments, or continuous-time models, to examine the timing of these processes.

Finally, the study focused on self-esteem and perceived social support. Other plausible pathways, including resilience, emotion regulation, school belonging, social identity, sleep, and selection into sports by psychologically healthier adolescents, were not examined. Future research should compare these competing mechanisms in preregistered full-panel or intervention designs.

## 5 Conclusions

Higher group-sport-related PARS-3 scores at baseline were prospectively associated with lower psychological distress at follow-up among adolescents in the participating schools. Self-esteem and perceived social support at T2 showed significant parallel indirect associations, with the self-esteem pathway being comparatively stronger. Exploratory conventional lagged analyses also showed an association from T1 self-esteem to T2 perceived social support, but this finding cannot be interpreted as a unidirectional within-person effect. Overall, the results are consistent with psychological and social pathways linking sport-related physical activity with adolescent mental health; however, convenience sampling, the general nature of PARS-3, asymmetric measurement, residual confounding, unequal lags, and the absence of an RI-CLPM preclude strong causal conclusions. Replication using probability-based multisite samples, group-sport-specific measures, complete multiwave panels, and intervention designs is warranted.

## Data Availability

The original contributions presented in the study are included in the article/Supplementary material, further inquiries can be directed to the corresponding author.
